# Factors That Impact Child Visiting in Adult Critical Care Settings—A Critical Literature Review

**DOI:** 10.1111/nicc.70660

**Published:** 2026-09-24

**Authors:** Charlotte Whittaker, Lucy Mottram, Hannah McCall

**Affiliations:** ^1^ School of Health & Social Care (Adult Nursing Team) Sheffield Hallam University Sheffield UK

**Keywords:** child visitors, critical care visiting, family engagement, family‐centred care, rights‐based considerations in health

## Abstract

**Background:**

Admission to adult critical care is highly stressful for patients and families. Regular visitation is associated with reduced delirium, improved satisfaction and lower anxiety among relatives. However, UK visiting policies remain inconsistent, with child visitors often restricted due to concerns about noise, supervision and infection risks.

**Aims:**

To examine factors influencing children visiting relatives in adult critical care, their psychological and emotional impacts, and the evidence underpinning current practices to inform UK guidance.

**Study Design:**

A critical literature review appraised English‐language, primary research studies (2010–2026) from CINAHL Complete and MEDLINE. Quantitative and qualitative studies involving children, adolescents, adults and healthcare professionals were included. Data were independently extracted by two researchers, appraised using the Mixed Methods Appraisal Tool (MMAT) and synthesised using descriptive, thematic analysis.

**Results:**

Eleven studies from the United Kingdom, the United States and Europe met inclusion criteria, including qualitative, survey, observational and quasi‐experimental designs. Methodological quality was moderate to high; however, non‐probability single‐site samples, limited control of confounding, small sample sizes and wide age ranges in child participants require cautious interpretation. Nurses frequently acted as gatekeepers, with visiting decisions often shaped more by personal beliefs than by evidence, although higher educational attainment was associated with more supportive attitudes towards child visiting. Parents sometimes withheld information to protect children; however, children generally wished to be included and reported less distress and greater self‐esteem following supported visits. Preparation and follow‐up helped make the critical care environment less frightening and more comprehensible.

**Conclusions:**

Supported child visiting appears beneficial and aligns with rights‐based, family‐centred care. Persistent barriers underline the need for evidence‐based policy and implementation.

**Relevance to Clinical Practice:**

UK critical care services should develop child‐centred visiting policies, implement structured visitor care plans, update the Step Competency Framework, and integrate child specialists into care delivery.

## Impact Statements

- What is known about the topic
  ○ Child visiting in adult critical care is inconsistent and is often shaped by nurses' beliefs, institutional culture and parental protection, despite evidence that supported visits can reduce children's anxiety and improve understanding.
- What this paper adds
  ○ This review highlights persistent barriers, clarifies the gatekeeping role of nurses, strengthens rights‐based arguments for inclusion and identifies practical priorities for policy, education and care planning.

## 1 Introduction

Admission to critical care is highly stressful for patients and relatives [1]. Regular visitation reduces patient delirium, improves satisfaction and can lessen relatives' anxiety and depression [2]. Despite this, critical care visiting policies in the United Kingdom remain inconsistent [3, 4].

Restrictive visiting practices can be traced back to 1800s almshouses, where stigma limited visitors [5]. Children were also historically discouraged from visiting due to concerns about noise, supervision and perceived patient distress [6]. Although existing evidence generally supports more open visiting [4], many UK critical care settings still restrict access for children [7].

## 2 Background/Justification for the Review

UK professional bodies currently offer only moderate support for child visiting. The Faculty of Intensive Care Medicine (FICM) promotes family‐centred care and encourages visiting but provides no implementation guidance (2024). Similarly, the British Association of Critical Care Nurses (BACCN) affirms patients' rights to visitors and calls for staff education to support child visiting, but its 2012 Position Statement was under review in 2023 and, to date, no revised statement has been published (2012).

Earlier reviews identified barriers to child visiting in adult intensive care [8, 9]. This review updates that evidence base by including more recent studies, including post–COVID‐19 publications, while excluding studies of COVID‐specific visiting practices.

## 3 Aim and Objectives

This review examined evidence relating to children visiting relatives in adult critical care to inform practice and support future guidance. The objectives were:

1. To identify the factors influencing whether and how children visit a critically ill relative in adult critical care settings.
2. To explore the reported psychological, emotional and social impact of visiting a critically ill relative on children.
3. To evaluate the evidence underpinning current practices and policies regarding child visitation in adult critical care environments.

## 4 Design and Methods

A critical literature review was undertaken in the United Kingdom to appraise and synthesise existing evidence [10].

### 4.1 Search Strategy

CINAHL Complete and MEDLINE were searched in March 2026. The following search string was developed using key word search terms, Boolean operators, truncation and phrase searching:

((Child* OR Adolescent* OR Youth OR Teenage* OR “Young Family”) NOT (Paediatric OR Pediatric)) AND (Visit* AND (“Critical Care” OR “CCU” OR “Intensive Therapy Unit” OR “ITU”)).

English‐language, full‐text studies primary research studies of any design were eligible if they addressed child visitors to adult critical care. Editorials and opinion pieces were excluded. Secondary sources, such as reviews and meta‐analyses, were also excluded because this review sought to focus on participants' direct accounts and original study data.

Studies focused on visiting during the COVID‐19 pandemic were excluded because suspended or altered visiting arrangements were not representative of routine practice [3, 11]. Studies of paediatric patients were also excluded. The date range was extended to 2010–2026 to yield enough literature for meaningful review [12].

### 4.2 Data Extraction and Analysis

Data extraction and quality appraisal were undertaken by two researchers (C.W. and H.M.). Reporting principles from PRISMA [13] were adapted for a critical literature review [10]. After repeated reading of each paper, data were extracted into a structured table of study characteristics (Table S1).

Methodological quality was appraised using the Mixed Methods Appraisal Tool (MMAT), version 2018 [14]. The MMAT was selected for this review as it is specifically designed to appraise the quality of qualitative, quantitative and mixed methods studies concurrently within a single tool, making it particularly suited for mixed studies reviews. The tool comprises two screening questions and up to 25 criteria organised across five study design categories; each category contains five core criteria rated as ‘Yes’, ‘No’ or ‘Can't tell’. Each study was screened and then assessed against five design‐specific criteria (Table S2). Quality appraisal was conducted independently by two reviewers (C.W. and H.M.) for each included study and disagreements were resolved through discussion. Quality appraisal results were used to inform the confidence of synthesis findings.

Data were analysed using descriptive thematic synthesis [15]. Findings were coded line‐by‐line and grouped inductively into four themes by a single researcher (C.W.): nurses' influence, parental influence, children's experiences, and preparation and follow‐up.

### 4.3 Ethical and Institutional Approval

This review analysed publicly available peer‐reviewed literature and did not require NHS or NIHR ethical approval. As a student project, it followed university ethics procedures and received institutional approval on 24/06/2024.

## 5 Findings

**FIGURE 1:**
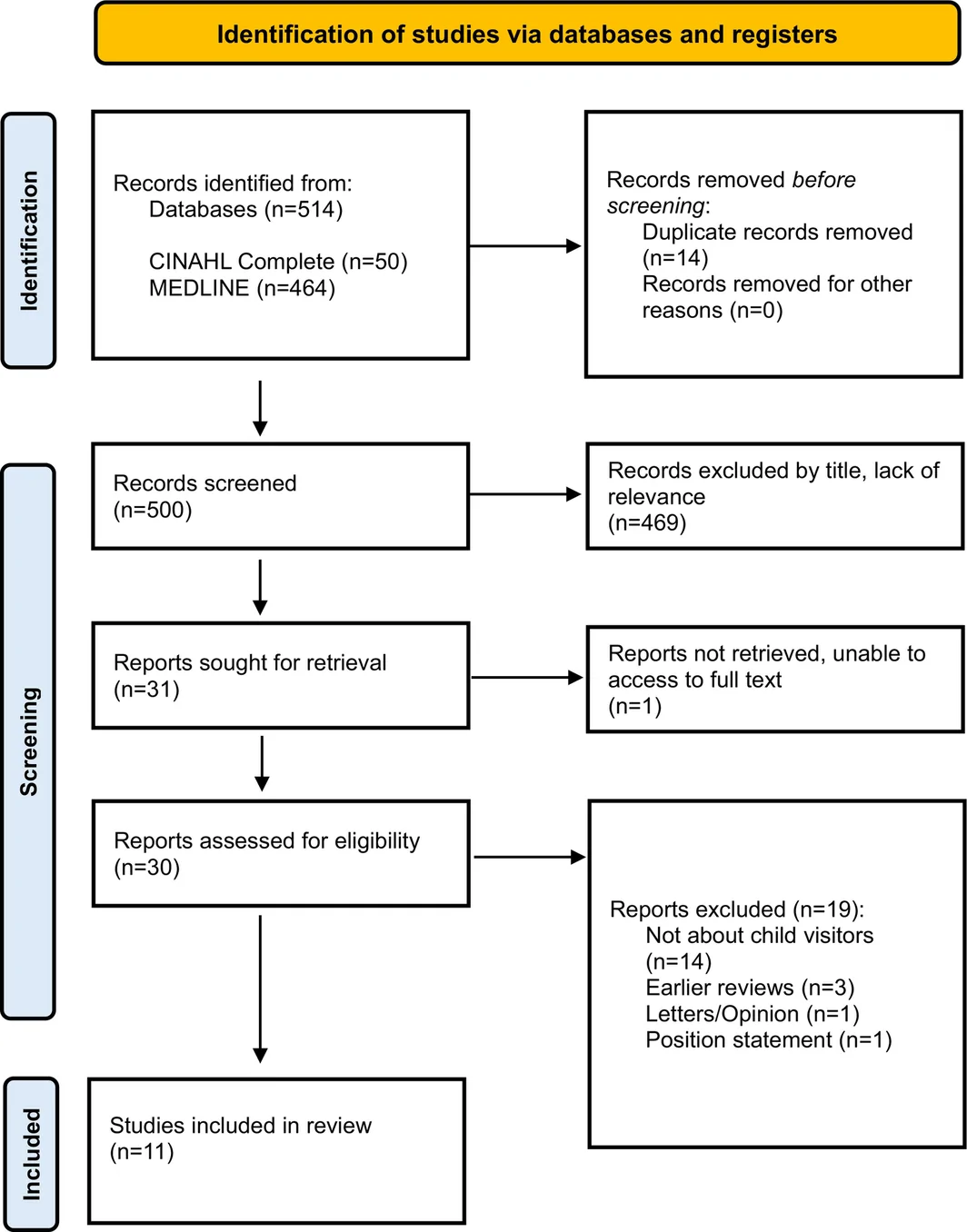
PRISMA diagram of literature search and report screening.

The search identified 514 records. After removal of 14 duplicates, 500 titles were screened, 31 abstracts reviewed and 30 full texts assessed. Nineteen papers were excluded as irrelevant and one full text could not be retrieved. Eleven studies published between 2010 and 2025 were included (Figure 1).

The studies came from the United Kingdom, United States and Europe. Sample sizes ranged from 7 to 446 participants and included children, adults and healthcare professionals. Included designs were qualitative, survey, observational and quasi‐experimental. See Table S1 for a summary of the included studies characteristics.

Methodological quality was generally good to high. Most studies met all applicable MMAT criteria (10/11) and one met four of five (Table S2). The main limitations were non‐probability single‐site sampling, limited control of confounding in non‐randomised studies, small sample sizes and wide age ranges in child participants. All studies were retained in keeping with the inclusive aims of a critical literature review, but their findings were interpreted cautiously.

Four themes are discussed here: the nurses' influence, parental influence, children's experiences, and preparation and follow‐up. The terms ‘parent’ and ‘parental’ in this manuscript refer to the child's primary care givers and could include biological or adoptive parents, grandparents and other guardians. The perspective of the unwell person is notably absent from the existing literature.

### 5.1 Nurses' Influence

The strongest evidence concerned the role of the bedside nurse in facilitating child visiting although children's perceptions of nurses were mixed. Kean [16] reported that children had positive impressions of nurses and found reassurance in the high nurse‐to‐patient ratio. Conversely, Knutsson et al. [17] found that some children saw nurses as caring but superficial, and not always good listeners or clear sources of information. Frivold et al. [18] found that children were not always given information directly by nurses, and more than half of Finnish respondents (56%) reported that they never provided information to child visitors. Knutsson and Bergbom [19] described nurses as ‘gatekeepers’, who had control over whether children could visit or not.

From the nurses' perspective, decisions about child visiting were often shaped by personal beliefs and feelings rather than evidence. Desai et al. [20] and Frivold et al. [18] both reported this pattern, and Valls‐Matarín et al. [21] found that staff applied differing rules according to the child's age and relationship to the patient. Infection control concerns were common: around half of nurses in Desai et al. [20] believed children were at risk from infection (*n* = 189/446) or posed a risk to patients (*n* = 201/446), and similar views were reported in Spain [21]. However, the reviewed literature did not support these beliefs with evidence of increased infection risk.

There were also examples of evidence‐informed practice. Knutsson et al. [22] found that some nurses had been persuaded by evidence‐based guidance that children should be included when a parent or relative was critically ill. These nurses believed visiting could reduce children's distress. Fergé et al. [23] similarly found that facilitating visits might reduce regret and lower the risk of post‐traumatic stress in adolescents. Desai et al. [20] and Frivold et al. [18] also found that nurses with higher levels of formal education were generally more open to child visiting and more aware of its benefits, although neither study explored causation.

### 5.2 Parental Influence

Parents and relatives often wanted to protect children from uncertainty and distress. Knutsson et al. [22] found that adults sometimes kept children away from critical care in the hope of preventing psychological harm. Belser et al. [24] reported from their small study of six participants that parents also felt discouraged by staff, which influenced their decisions. The desire to protect can also extend to limiting what children are told. In another small study, with seven participants, Knutsson et al. [17] found that children sometimes felt adults did not fully answer their questions.

However, multiple papers found that tailored visiting policies may reduce children's distress [18, 19, 22, 23], and well‐supported visits could create shared experiences that strengthen family bonds [24, 25].

### 5.3 Children's Experiences

Across several studies, children expressed a strong desire to be present, included, and needed. At the same time, they often felt forgotten or overlooked by adults.

Knutsson and Bergbom's [19] study of 28 participants found that although visiting could be upsetting, the need to see the unwell relative was usually more important than fear. Kean [16] showed that seeing their relative in critical care helped children make sense of the situation. Knutsson et al. [17] found that physical closeness, although initially frightening, often reduced distress.

Some children also described improved confidence and self‐esteem after visiting. In Knutsson et al. [17], children felt ‘helpful’ and ‘proud’ to show care and compassion. Once familiar with the environment, they often wanted to reach their relative quickly and stay as long as possible. In contrast, adolescents who did not visit were more likely to report regret [23, 25, 26].

### 5.4 Preparation and Follow‐Up

Five papers argued explicitly that, with appropriate support, child visiting in critical care can be positive for the child, patient and family.

Kean [16] found that children were often curious about beside technology, and Knutsson and Bergbom [19] found that children could understand their relative's dependence on machines while in a critical care setting. Knutsson et al. [17] reported that explanation and demonstration of equipment could improve understanding. However, Fergé et al. [25] found that most adolescents in their study felt they had not received enough information (80%, 42/53), and Desai et al. [20] reported that most nurses lacked child‐friendly resources (88.6%, 395/446).

Several studies highlighted the value of individualised planning, with information tailored to the family's circumstances and the child's age and needs [17, 19, 21, 25, 26]. Educational booklets were suggested as a way to facilitate open communication and prepare children for the visit, while dedicated child‐friendly spaces with toys and books were also recommended [24, 25, 26].

Some studies discussed a possible role for child specialists, including school counsellors, school nurses and child life specialists. These professionals could support preparation and follow‐up, but none of the included studies described child follow‐up as routine practice [17, 18, 20, 25].

### 5.5 Summary of Key Findings

This review found that child visiting in adult critical care is shaped more by nurses' beliefs, parental efforts to protect children and inconsistent local practice than by robust evidence. Four patterns were clear: nurses frequently act as gatekeepers, with decisions often influenced by experience and assumptions rather than evidence; parents may try to protect children by withholding information; children usually want to be included and often benefit from visiting; and structured preparation and follow‐up may make the environment easier for children to understand and manage.

Although the evidence for these findings is often supported by small studies, the same patterns recurred across the reviewed literature, strengthening transferability and generalisability within the findings.

## 6 Discussion

The findings from studies in the United Kingdom, United States and Europe suggest that decisions about child visiting in adult critical care are still shaped more by individual attitudes and local norms than by child‐led, research‐informed practice.

The nurse emerged as both facilitator and gatekeeper. This is consistent with wider literature that positions nurses as central to visiting practices because of their close involvement with patients and families and their responsibility for safety within clinical environments [9, 27]. Consensus guidance from the German Interdisciplinary Association of Critical Care and Emergency Medicine emphasises that clinicians should critically reflect on their own beliefs as gatekeepers and develop policies that embed child visits into family‐centred care, recognising that restricting access without clear harm is inconsistent with contemporary critical care values [28]. Lamiani et al. [26] further suggest that staff require training to understand children's needs and to manage their presence in adult critical care.

In the United Kingdom, professional bodies support flexible, family‐centred visiting, but there is little detailed child‐specific guidance [29, 30]. Visiting, of any kind, is not explicitly mentioned in current UK health regulations [31] and national public‐facing guidance on visiting critical care units is non‐specific [7]. The Department of Health & Social Care requires that healthcare professionals ensure their practice is ‘person‐centred’ and ‘meets their [patients] needs and preferences’ without specifying how this should be achieved [3]; while the UK's National Institute for Health and Care Excellence (NICE) guidance promotes family involvement following critical illness, but does not discuss children directly (2009; and 2017). This lack of detailed national guidance may be contributing to inconsistent implementation of child‐visiting practice.

The influence of the well parent or relative was another important factor. Although protective instincts are understandable and often well‐intentioned, both the findings of this review and wider literature suggest that withholding information can increase children's anxiety and isolation. Kean [32] describes such children as ‘silent listeners’, trying to gather information indirectly without fully understanding what they hear. By contrast, involving children can help maintain family attachment and improve understanding [28].

The evidence also supports the importance of children's own experiences. A systematic review by Lamiani et al. [33] found that visits can be distressing but also help children understand reality and preserve relationships with relatives. The improvements in confidence, self‐esteem and sense of contribution reported in several included studies challenge the assumption that critical care settings are inherently harmful to children [17, 19, 24, 25, 26].

There are also legal and ethical implications in excluding children without clear justification. The United Nations Convention on the Rights of the Child [34] emphasises both children's right to express their views and their right to maintain family relationships, and blanket exclusion or restrictive visiting policies may conflict with these core principles. In the United Kingdom, the Human Rights Act 1998, Article 8, further protects the right to family life and within rights‐based healthcare, restrictions on visiting should be legitimate, proportionate and clearly justified [3].

Preparation and follow‐up remain important but underdeveloped aspects of practice. The findings support the view that preparation reduces fear by demystifying the environment and technology in critical care settings. International recommendations highlight age‐appropriate preparation, psychosocial support and follow‐up as central to child‐friendly visiting [28]. The lack of routine follow‐up is particularly striking given evidence linking critical illness experiences to later psychological outcomes [35]. Although international literature supports the role of child specialists, there is currently no routinely comparable role within UK adult critical care [9, 36].

Recent recommendations for bereaved children in adult critical care include advocacy for child visitors, clearer guidance and specialised staff training [37]. However, only approximately a fifth of admissions to critical care settings in the UK result in death [38], and the findings in this review suggest that such support should extend to all child visitors and not only those whose relative is dying.

A final gap in the literature is the absence of the unwell patient's perspective. Critically ill patients are often excluded from research because of concerns about consent, communication and burden [39, 40]. The resulting knowledge gap represents a missed opportunity to gain a fully holistic understanding of what family‐centred care could look like, especially given evidence that family presence may support patient motivation and emotional wellbeing [2, 41].

Overall, this review confirms that many issues identified in earlier reviews remain unresolved. When carefully supported, child visiting appears more often beneficial than harmful and is consistent with contemporary best practice and human rights principles. Moving practice forward requires a shift away from discretionary, belief‐driven decisions towards structured, evidence‐based and ethically grounded approaches.

## 7 Limitations

This review has several limitations. Although data extraction and quality appraisal were undertaken by two researchers, the theme development was completed by a single reviewer, which increases the risk of subjective judgement at this stage. This risk was reduced through repeated reading, a structured extraction form, standardised MMAT criteria, and transparent reporting, but independent double coding and subsequent collaborative theme development would have strengthened rigour.

The review was not pre‐registered, and the extraction form was not formally piloted. Generalisability is also limited by the small evidence base, English‐language restriction and the inclusion of studies from health systems that differ from the United Kingdom. However, the consistency of findings across studies and their alignment with emerging guidance suggest some transferability.

## 8 Implications for Practice

1. *Develop standardised, child‐centred visiting policies*: Policies should present child visiting as a normal part of family‐centred critical care and uphold children's rights.
2. *Use structured care plans for child visitors*: Preparation, age‐appropriate explanation, planned follow‐up and active staff engagement should begin early in admission.
3. *Revise the step competency framework*: The UK framework [42] should include explicit education on supporting child visitors in critical care.
4. *Integrate child‐specific expertise into teams*: Child life specialists or similar roles could support families and staff and improve children's inclusion.
5. *Set future research priorities*: Further research is needed on critically ill patients' views, the longer‐term effects of visiting or not visiting, the effectiveness of preparation and follow‐up, and the development of child‐friendly resources. Comparing pre– and post–COVID‐19 pandemic visiting policies and attitudes is also a valuable direction for future research.

## 9 Conclusion

Supported visits were generally associated with reduced anxiety, better understanding, improved self‐esteem and stronger family connection for children, while the main barriers were staff gatekeeping, inadequate information sharing and the lack of child‐specific preparation and follow‐up.

Routine exclusion of children is difficult to justify when visits could be carefully planned and supported. Current UK guidance remains too vague to ensure consistent child‐centred practice, leaving decisions vulnerable to local custom rather than clear evidence or rights‐based principles.

The evidence base is still limited by small studies, English‐language restriction and the predominance of research from outside the United Kingdom, which reduces generalisability. The absence of the critically ill patient's perspective is another important gap. Overall, the review supports a shift towards structured, evidence‐based and ethically grounded child‐visiting policies in adult critical care, supported by clearer guidance, staff education and age‐appropriate preparation and follow‐up.

## Funding

The authors have nothing to report.

## Ethics Statement

This literature review did not require NIHR or NHS ethical approval as it involved analysis of publicly available, peer‐reviewed literature only. However, as a student project, Sheffield Hallam University's Research Ethics Policy and Procedures (2020) were followed, and the University's internal ethics review process was followed. Internal ethics approval was granted by the University on 24/06/2024.

## Consent

The authors have nothing to report.

## Conflicts of Interest

The authors declare no conflicts of interest.

## Supporting information


**Table S1:** Summary characteristics of all included studies, with abbreviated MMAT quality score.

**Table S2:** Item‐level MMAT quality appraisal ratings for all included studies.

## Data Availability

The data that support the findings of this study are available from the corresponding author upon reasonable request.
