# Supplementary material for: Factors That Impact Child Visiting in Adult Critical Care Settings—A Critical Literature Review
Source: Nurs Crit Care. 2026 Sep 24;31(6):e70660. doi: 10.1111/nicc.70660 (PMC13612940; doi:10.1111/nicc.70660)
Supplement: Supplementary file 1 [file NICC-31-0-s001.docx]

**Supplementary File 2**

**Table 2:** Item-level MMAT quality appraisal ratings for all included studies

|  | **Study** | **Design Type** | **Screening Q1** | **Screening Q2** | **1.1** | **1.2** | **1.3** | **1.4** | **1.5** | **2.1** | **2.2** | **2.3** | **2.4** | **2.5** | **3.1** | **3.2** | **3.3** | **3.4** | **3.5** | **4.1** | **4.2** | **4.3** | **4.4** | **4.5** | **5.1** | **5.2** | **5.3** | **5.4** | **5.5** | **Overall rating** |
| --- | --- | --- | --- | --- | --- | --- | --- | --- | --- | --- | --- | --- | --- | --- | --- | --- | --- | --- | --- | --- | --- | --- | --- | --- | --- | --- | --- | --- | --- | --- |
|  |  |  |  |  | **Qualitative** | | | | | **Randomised controlled trials** | | | | | **Non-randomised study** | | | | | **Quantitative descriptive** | | | | | **Mixed methods** | | | | |  |
| **1** | Kean (2010) | Qualitative | **Y** | **Y** | **Y** | **Y** | **Y** | **Y** | **Y** | - | - | - | - | - | - | - | - | - | - | - | - | - | - | - | - | - | - | - | - | **5/5** |
| **2** | Knutsson & Bergbom (2016) | Qualitative | **Y** | **Y** | **Y** | **Y** | **Y** | **Y** | **Y** | - | - | - | - | - | - | - | - | - | - | - | - | - | - | - | - | - | - | - | - | **5/5** |
| **3** | Knutsson *et al*. (2017) | Qualitative | **Y** | **Y** | **Y** | **Y** | **Y** | **Y** | **Y** | - | - | - | - | - | - | - | - | - | - | - | - | - | - | - | - | - | - | - | - | **5/5** |
| **4** | Fergé *et al*. (2018) | Quantitative, non-randomised | **Y** | **Y** | - | - | - | - | - | - | - | - | - | - | **Y** | **Y** | **Y** | **Y** | **Y** | - | - | - | - | - | - | - | - | - | - | **5/5** |
| **5** | Desai *et al*. (2020) | Quantitative, non-randomised | **Y** | **Y** | - | - | - | - | - | - | - | - | - | - | **Y** | **Y** | **Y** | **Y** | **Y** | - | - | - | - | - | - | - | - | - | - | **5/5** |
| **6** | Valls-Matarín *et al*. (2020) | Quantitative, non-randomised | **Y** | **Y** | - | - | - | - | - | - | - | - | - | - | **Y** | **Y** | **Y** | **Y** | **Y** | - | - | - | - | - | - | - | - | - | - | **5/5** |
| **7** | Fergé *et al*. (2021) | Quantitative, non-randomised | **Y** | **Y** | - | - | - | - | - | - | - | - | - | - | **Y** | **Y** | **Y** | **Y** | **Y** | - | - | - | - | - | - | - | - | - | - | **5/5** |
| **8** | Knutsson *et al*. (2021) | Qualitative | **Y** | **Y** | **Y** | **Y** | **Y** | **Y** | **Y** | - | - | - | - | - | - | - | - | - | - | - | - | - | - | - | - | - | - | - | - | **5/5** |
| **9** | Frivold *et al*. (2022) | Quantitative, non-randomised | **Y** | **Y** | - | - | - | - | - | - | - | - | - | - | **Y** | **Y** | **Y** | **Y** | **Y** | - | - | - | - | - | - | - | - | - | - | **5/5** |
| **10** | Belser *et al*. (2024) | Qualitative | **Y** | **Y** | **Y** | **Y** | **Y** | **Y** | **Y** | - | - | - | - | - | - | - | - | - | - | - | - | - | - | - | - | - | - | - | - | **5/5** |
| **11** | Lamiani *et al*. (2025) | Quantitative, non-randomised | **Y** | **Y** | - | - | - | - | - | - | - | - | - | - | **Y** | **Y** | **CT** | **Y** | **Y** | - | - | - | - | - | - | - | - | - | - | **4/5** |

| **Rating codes:** | Y = Yes | N = No | CT = Can't Tell | (-) = Not applicable |  |
| --- | --- | --- | --- | --- | --- |
| **Scoring key:** | 5/5 criteria met (100%) | 4/5 met (80%) | 3/5 met (60%) | 2/5 met (40%) | 1/5 met (20%) |

**Notes**

- MMAT = Mixed Methods Appraisal Tool, version 2018 (Hong *et al.*, 2018).
- Each criterion was independently rated by two reviewers (CW and HM) as 'Yes' (Y), 'No' (N), or 'Can't tell' (CT). Only the criterion categories applicable to each study's design were rated; non-applicable criteria are indicated with (-). Full criteria definitions are available in the MMAT 2018 user guide (Hong *et al.*, 2018).
