# Supplementary material for: Factors That Impact Child Visiting in Adult Critical Care Settings—A Critical Literature Review
Source: Nurs Crit Care. 2026 Sep 24;31(6):e70660. doi: 10.1111/nicc.70660 (PMC13612940; doi:10.1111/nicc.70660)
Supplement: Supplementary file 2 [file NICC-31-0-s002.docx]

**Supplementary File 1**

**Table 1:** Summary characteristics of all included studies, with abbreviated MMAT quality score

|  | **Study** | **Country** | **Population** | **Age range of children** | **Study design** | **Sample size** | **Data collection method** | **MMAT category** | **MMAT quality criteria met** |
| --- | --- | --- | --- | --- | --- | --- | --- | --- | --- |
| **1** | Kean (2010) | UK (Scotland) | Families with children/young people, with a family member in adult ICU | 8-25 years | Constructivist grounded theory qualitative study | 9 families; 24 participants  (12 adults and 12 children/adolescents) | Family group interviews | Qualitative | **5/5** |
| **2** | Knutsson & Bergbom (2016) | Sweden | Children & adolescents with a family member in adult ICU | 4-17 years | Qualitative descriptive study | 28 children & adolescents | Individual interviews & drawings | Qualitative | **5/5** |
| **3** | Knutsson *et al.* (2017) | Sweden | Adult ICU nurses | Not applicable | Qualitative exploratory study | 23 nurses; in 4 focus groups | Focus groups | Qualitative | **5/5** |
| **4** | Fergé *et al.* (2018) | France | Adolescents with a family member in adult ICU | 12-17 years | Prospective observational study | 53 adolescents | Questionnaires | Quantitative non-randomised | **5/5** |
| **5** | Desai *et al.* (2020) | USA | Adult ICU nurses | Not applicable | Cross-sectional survey | 446 nurses | Questionnaires | Quantitative non-randomised | **5/5** |
| **6** | Valls-Matarín *et al.* (2020) | Spain | Adult ICU healthcare staff | Not applicable | Descriptive cross-sectional study | 62 staff; 31 nurses, 13 nursing auxiliaries & 18 doctors | Questionnaires | Quantitative non-randomised | **5/5** |
| **7** | Fergé *et al.* (2021) | France | Adolescents with a family member in adult ICU | 12-17 years | Longitudinal prospective observational study | 46 adolescents | Questionnaires | Quantitative non-randomised | **5/5** |
| **8** | Knutsson *et al.* (2021) | Sweden | Children & adolescents with a family member in adult ICU | 6-18 years | Qualitative, descriptive phenomenological study | 3 families; 7 children & adolescents | Individual interviews, drawings & questionnaires | Qualitative | **5/5** |
| **9** | Frivold *et al.* (2022) | Norway, Denmark, Sweden, Finland | Adult ICUs | Not applicable | Cross-sectional survey | 158 adult ICUs; 1 nurse or nurse coordinator per department | Questionnaires | Quantitative non-randomised | **5/5** |
| **10** | Belser *et al.* (2024) | Switzerland | Parents* of children/young people, with a family member in adult ICU | Not applicable | Qualitative, descriptive study | 6 adult parents, of children aged between 5 months and 17 years | Semi-structured interviews | Qualitative | **5/5** |
| **11** | Lamiani *et al.* (2025) | Italy | Children & adolescents with a family member in adult ICU | 7-17 years | Quasi-experimental, pre-post intervention study | 25 children & adolescents | Questionnaires | Quantitative non-randomised | **4/5** |

**Notes**

- ICU = Intensive care unit.
- MMAT = Mixed Methods Appraisal Tool, version 2018 (Hong *et al*., 2018).
- *Parent/s = The term “parent” refers to the child's primary care givers and could include biological or adoptive parents, grandparents, and other guardians.
- Quality appraisal was conducted independently by two reviewers (CW and HM); disagreements were resolved by discussion. Full item-level ratings and criterion notes are presented in Supplementary File 2.
